# A non-randomized risk-adjusted comparison of lenalidomide + R-CHOP versus R-CHOP for *MYC*-rearranged DLBCL patients

**DOI:** 10.1038/s41408-023-00854-2

**Published:** 2023-05-22

**Authors:** A. Vera de Jonge, Erik van Werkhoven, Avinash G. Dinmohamed, Marcel Nijland, Aeilko H. Zwinderman, Patrick M. Bossuyt, Martine S. Veldhuis, Emma G. G. M. Rutten, Rogier Mous, Joost S. P. Vermaat, Yorick Sandberg, Eva de Jongh, Yavuz M. Bilgin, Rinske Boersma, Harry Koene, Marie José Kersten, Daphne de Jong, Martine E. D. Chamuleau

**Affiliations:** 1 Department of Hematology, Amsterdam UMC location VU, Amsterdam, The Netherlands; 2 HOVON Data Center, Department of Hematology, Erasmus MC Cancer Institute, Rotterdam, The Netherlands; 3 Erasmus MC, Department of Public Health, University Medical Center Rotterdam, Rotterdam, The Netherlands; 4 Department of Research and Development, Netherlands Comprehensive Cancer Organisation (IKNL), Utrecht, The Netherlands; 5 Department of Hematology, University Medical Center Groningen, Groningen, The Netherlands; 6 Department of Clinical Epidemiology, Biostatistics, and Bioinformatics, Amsterdam UMC, University of Amsterdam, Amsterdam, The Netherlands; 7 Department of Pathology, Amsterdam UMC location VU, Amsterdam, The Netherlands; 8 Department of Hematology, University Medical Center Utrecht, Utrecht, The Netherlands; 9 Department of Hematology, Leiden University Medical Center, Leiden, The Netherlands; 10 Department of Internal Medicine, Maasstad Hospital, Rotterdam, The Netherlands; 11 Department of Hematology, Albert Schweitzer Ziekenhuis, Dordrecht, The Netherlands; 12 Department of Internal Medicine, Adrz, Goes, The Netherlands; 13 Department of Internal Medicine, Amphia Ziekenhuis, Breda, The Netherlands; 14 Department of Hematology, St Antonius Ziekenhuis, Nieuwegein, The Netherlands

**Keywords:** Epidemiology, B-cell lymphoma, B-cell lymphoma

## Abstract

Patients with *MYC* rearranged (*MYC*-R) diffuse large B-cell lymphoma (DLBCL) have a poor prognosis. Previously, we demonstrated in a single-arm phase II trial (HOVON-130) that addition of lenalidomide to R-CHOP (R2CHOP) is well-tolerated and yields similar complete metabolic remission rates as more intensive chemotherapy regimens in literature. In parallel with this single-arm interventional trial, a prospective observational screening cohort (HOVON-900) was open in which we identified all newly diagnosed *MYC*-R DLBCL patients in the Netherlands. Eligible patients from the observational cohort that were not included in the interventional trial served as control group in the present risk-adjusted comparison. R2CHOP treated patients from the interventional trial (*n* = 77) were younger than patients in the R-CHOP control cohort (*n* = 56) (median age 63 versus 70 years, *p* = 0.018) and they were more likely to have a lower WHO performance score (*p* = 0.013). We adjusted for differences at baseline using 1:1 matching, multivariable analysis, and weighting using the propensity score to reduce treatment-selection bias. These analyses consistently showed improved outcome after R2CHOP with HRs of 0.53, 0.51, and 0.59, respectively, for OS, and 0.53, 0.59, and 0.60 for PFS. Thus, this non-randomized risk-adjusted comparison supports R2CHOP as an additional treatment option for *MYC*-R DLBCL patients.

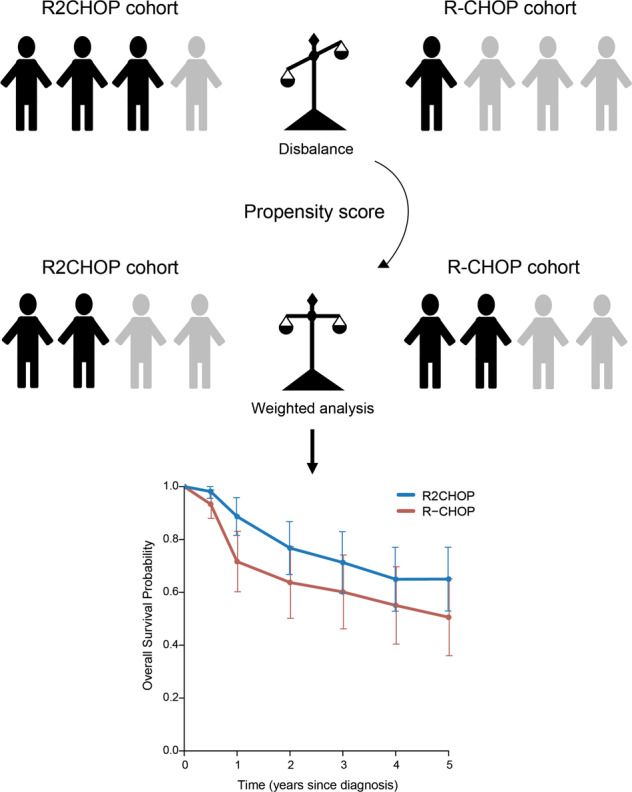

## Introduction

First-line immunochemotherapy with rituximab, cyclophosphamide, doxorubicin, vincristine, and prednisone (R-CHOP) cures the majority of diffuse large B-cell lymphoma (DLBCL) patients [1, 2]. The most commonly used prognostic score is the International Prognostic Index (IPI), which consists of age (>60 years), Ann-Arbor stage (III/IV), WHO performance score (≥2), lactate dehydrogenase (LDH) serum level (elevated), and number of extra-nodal localizations (>1) [3, 4]. Other well-known prognostic disease characteristics are sex [5], cell-of-origin (COO) [6] and the presence of a rearrangement of the *MYC* oncogene (normally located on chromosome 8q24.21), which is detected in 10–15% of all newly diagnosed DLBCL cases [7]. Compared with a 5-year overall survival (OS) of 72% and 5-year progression-free survival (PFS) of 66% in patients without a *MYC*-rearrangement, *MYC*-rearranged (*MYC*-R) patients have a 5-year OS and PFS of 33% and 31%, respectively [8]. In a more recent study, *MYC*-R patients had a 5-year OS of 49% [9].

In 70% of *MYC*-R patients a *MYC* rearrangement is detected with a concomitant *BCL2* (located on chromosome 18q21.33) or *BCL6* (chromosome 3q27.3) rearrangements (double hit [DH]), or with both *BCL2* and *BCL6* rearrangements (triple hit [TH]) [10]. The remaining 30% of the patients only have a *MYC* rearrangement only (single hit [SH]) [7]. The inferior prognosis of a *MYC* rearrangement is largely attributed to patients with a DH/TH lymphoma [7] and, therefore, these subsets have been defined as a separate entity since 2016 [11].

Intensified immunochemotherapy regimens have been investigated to improve first-line treatment for *MYC*-R patients. Such regimens, e.g., hyper-CVAD and R-CODOX-M/R-IVAC, seemed to improve survival, but only evaluated in retrospective studies [9, 12]. In a prospective study, dose-adjusted EPOCH-R (DA-EPOCH-R) showed promising complete metabolic remission (CMR) rates of 74% at end of treatment and resulted in a 4-year event-free survival (EFS) of 71% and OS of 77% for all *MYC-*R patients [13]. DH/TH patients had an even better EFS of 73% and OS of 82% [13]. Based on this study, many groups worldwide consider DA-EPOCH-R as the preferred first-line regimen for *MYC*-R patients, especially for DH/TH patients.

Other strategies to improve outcome for *MYC*-R DLBCL patients have focused on addition of novel drugs to the R-CHOP backbone. For example, in the CAVALLI phase II study, the selective BCL2 inhibitor venetoclax was added to R-CHOP showing promising results, especially in DH lymphomas with high levels of BCL2 protein expression [14]. Adding venetoclax to DA-EPOCH-R, however, turned out to be too toxic, resulting in early discontinuation of the subsequent phase III randomized study in DH lymphomas [15].

The rationale for adding lenalidomide to the R-CHOP backbone for *MYC*-R DLBCL is the MYC-downregulating effect of lenalidomide via cereblon targeting [16, 17]. In a single-arm phase II trial for newly diagnosed *MYC*-R patients (‘HOVON-130’), we have shown that addition of lenalidomide to R-CHOP is well-tolerated and resulted in a complete metabolic remission (CMR) in 67% of patients at end of treatment and a 2-year OS and EFS of 73% and 63%, respectively [18]. Here, we have selected a cohort of *MYC*-R patients from a simultaneously open, prospective population-based registration cohort of R-CHOP-treated DLBCL patients (HOVON-900 cohort) as controls to compare with the long-term follow-up data of the R2CHOP interventional group (HOVON-130 trial [18]). In this comparison, we use three statistical models (1:1 matching of the groups on IPI score, multivariable analysis and propensity score weighting) to assess the added value of lenalidomide to R-CHOP in terms of OS and PFS.

## Methods

### Patient selection

In the HOVON-130 trial, *MYC*-R DLBCL patients ≥18 years were treated with R-CHOP21 plus lenalidomide 15 mg day 1–15 for 6 cycles [18]. Additional inclusion criteria were Ann-Arbor stage II-IV, WHO performance status 0–3, ≥ one lesion of ≥1.5 cm on contrast-enhanced CT scan and ≥ one FDG-positive lesion on PET-CT scan. Exclusion criteria were: other subtype of aggressive B-cell lymphoma, history of follicular lymphoma, proven CNS localization, or HIV infection.

Concurrent with the HOVON-130 trial (2015–2019), the HOVON-900 observational protocol was open for newly diagnosed *MYC*-R DLBCL patients in the Netherlands [19]. *MYC*, *BCL2* and *BCL6* fluorescent in situ hybridization (FISH) diagnostics were advocated as part of routine procedures and reviewed by the HOVON Pathology Facility.

We selected all HOVON-900 newly diagnosed *MYC*-R DLBCL patients treated with R-CHOP who met all inclusion criteria of the HOVON-130 trial. Patients with a transformed lymphoma or history of follicular lymphoma were not included. Baseline data and routinely collected outcome data were retrieved from the Netherlands Cancer Registry (NCR).

According to the Central Committee on Research Involving Human Subjects in the Netherlands (CCMO), this type of observational study does not require ethics committee approval. The use of anonymous data for this study has been approved by the Privacy Review Board of the NCR.

### Statistical methods

Overall survival (OS) and progression-free survival (PFS) were calculated from date of diagnosis to death (OS) and to relapse or death (PFS), censoring patients without event. The Kaplan–Meier method and Cox regression were used for unadjusted analysis of OS and PFS.

We explored three statistical methods that account for baseline imbalances: matching, multivariable regression, and inverse probability of treatment weighting (IPTW) using a propensity score.

First, we performed one-to-one matching on the IPI risk score (low, intermediate, or high), because it is the most widely used and validated prognostic score. The HR from this analysis estimates the treatment effect on patients treated with R2CHOP in this particular sample. As patients without match were excluded for this analysis, we additionally used two other statistical methods that make use of the entire cohorts: multivariable regression and inverse probability of treatment weighting (IPTW).

We used multivariable regression as a second method to adjust for the individual variables of the IPI score (age, Ann Arbor stage, number of extra-nodal localizations, LDH serum levels, and WHO performance status) and rearrangement status (SH versus DH/TH) because these are known prognostic factors for overall survival. The resulting HR is an adjusted HR.

Thirdly we performed IPTW using a propensity score. The resulting HR is most likely to reflect what would have been observed in an unadjusted randomized comparison, in contrast to matching and multivariable analysis. We calculated a propensity score for being included in the HOVON-130 trial based on the separate components of the IPI score (see above). We additionally included sex and rearrangement status (single hit versus double/triple hit). To allow for some degree of misspecification of the model for the propensity score, we did a separate analysis using a doubly robust estimator to obtain absolute estimates.

## Results

### Patients

**Fig. 1:**
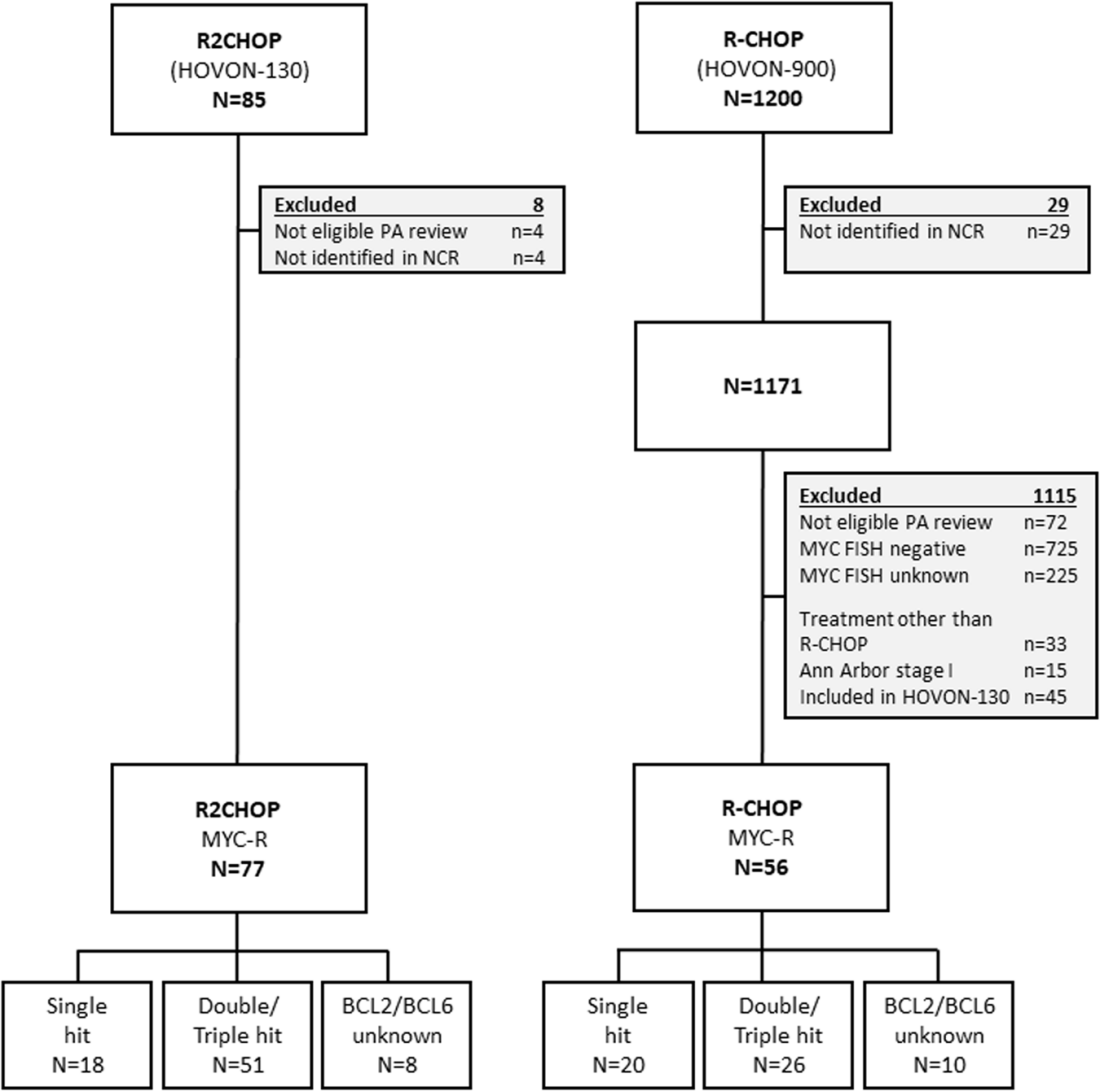
Flow chart of patient selection. Flow chart of the patients included in the HOVON-130 and HOVON-900 for the current comparison.

Of the 85 patients enrolled in the interventional R2CHOP cohort (HOVON-130 trial), 8 patients were ineligible for the present analysis (three because a *MYC* translocation could not be confirmed, and one because of transformed synchronous follicular lymphoma and four could not be identified in the NCR database). Data of 1171 (98%) of the 1200 DLBCL patients registered in the observational HOVON-900 cohort could be retrieved from the NCR. Of these, 1022 patients were excluded due to ineligible PA review or negative/unknown MYC FISH status and 45 patients were already included in the HOVON-130 trial. An additional 48 patients did not meet the eligibility criteria (i.e., 17 patients received no treatment at all, 16 patients were not treated with R-CHOP, but with another regimen (Table S1) and an additional 15 patients had an Ann Arbor stage I). Eventually, 56 (4.7% of 1171) fulfilled the eligibility criteria to serve as control in the present study (Fig. 1). Reasons for not being included in the interventional R2CHOP trial despite meeting its inclusion criteria were mainly logistic, e.g., the trial was not open in that center at that time, or the patient did not want to be referred or to participate.

Patients in the R2CHOP cohort received treatment between April 2015 and February 2018, and patients in the R-CHOP cohort between August 2015 and June 2019. Median follow-up was 4.16 years in patients treated with R2CHOP and 3.65 years in patients treated with R-CHOP (*p* = 0.87). The median time between diagnosis and start of treatment (diagnosis to treatment interval) was 19 days (range 0–69 days) in the R2CHOP group and 15 days (range 5–84 days) in the R-CHOP group (*p* = 0.317).

Various baseline characteristics were imbalanced between the cohorts (Table 1). Patients treated with R2CHOP were younger than patients treated with R-CHOP (median age 63 versus 70 years, *p* = 0.018), were more likely to have a lower WHO performance score (*p* = 0.013) and, as a consequence, had more often an intermediate IPI score (i.e., less often a low IPI score and less often a high IPI score, *p* = 0.004). There was no statistical proof that the distribution of sex, Ann Arbor stage, LDH levels, and rearrangement status were different between the cohorts, but there were numerical differences. For example, the R2CHOP group consisted of 18/77 SH patients (23.4%), 51/77 DH patients (66.2%), and in 8 patients (10.4%) *BCL2* and *BCL6* status were both missing. In the R-CHOP cohort 20/56 patients (35.7%) were SH, 26/56 patients (46.4%) were DH and in 10/56 patients (17.9%) *BCL2* and *BCL6* status were missing. Cell-of-origin status based on the Hans algorithm was not different between the two treatment groups, with the majority of the casus (80.5%) being germinal center B-cell (GCB) type DLBCL in both groups (*p* = 0.999).

**Table 1:** Baseline characteristics by treatment group.

	R-CHOP (*N* = 56)	R2CHOP (*N* = 77)	Total (*N* = 133)	*p*-value
Age at incidence (years)				0.018^a^
Median	70	63	66	
IQR	57–75	54–72	56–73	
Range	29–88	28–82	28–88	
Sex				0.271^b^
Male	34 (60.7%)	54 (70.1%)	88 (66.2%)	
Female	22 (39.3%)	23 (29.9%)	45 (33.8%)	
Ann Arbor stage				0.172^c^
2	12 (21.4%)	10 (13.0%)	22 (16.5%)	
3	12 (21.4%)	11 (14.3%)	23 (17.3%)	
4	32 (57.1%)	56 (72.7%)	88 (66.2%)	
WHO performance score				0.013^c^
0	22 (41.5%)	47 (61.0%)	69 (53.1%)	
1	16 (30.2%)	24 (31.2%)	40 (30.8%)	
2	10 (18.9%)	5 (6.5%)	15 (11.5%)	
3	5 (9.4%)	1 (1.3%)	6 (4.6%)	
(Missing)	3	0	3	
WHO PS (grouped)				0.006^c^
0	22 (41.5%)	47 (61.0%)	69 (53.1%)	
1	16 (30.2%)	24 (31.2%)	40 (30.8%)	
2 or 3	15 (28.3%)	6 (7.8%)	21 (16.2%)	
(Missing)	3	0	3	
LDH				0.693^b^
Within normal range	16 (28.6%)	19 (25.0%)	35 (26.5%)	
Elevated	40 (71.4%)	57 (75.0%)	97 (73.5%)	
(Missing)	0	1	1	
Extra-nodal localizations				0.300^c^
None	12 (21.4%)	23 (29.9%)	35 (26.3%)	
1	22 (39.3%)	21 (27.3%)	43 (32.3%)	
2 or more	22 (39.3%)	33 (42.9%)	55 (41.4%)	
IPI risk group				0.013^c^
Low	12 (21.8%)	9 (11.8%)	21 (16.0%)	
Low-intermediate	8 (14.5%)	22 (28.9%)	30 (22.9%)	
High-intermediate	13 (23.6%)	29 (38.2%)	42 (32.1%)	
High	22 (40.0%)	16 (21.1%)	38 (29.0%)	
(Missing)	1	1	2	
IPI Risk (3 Groups)				0.004^c^
Low	12 (21.8%)	9 (11.7%)	21 (15.9%)	
Intermediate	21 (38.2%)	52 (67.5%)	73 (55.3%)	
High	22 (40.0%)	16 (20.8%)	38 (28.8%)	
(Missing)	1	0	1	
COO IHC (Hans classification)				0.999^b^
GCB subtype	45 (80.4%)	62 (80.5%)	107 (80.4%)	
Non-GCB subtype	5 (8.9%)	8 (10.3%)	13 (9.8%)	
Not evaluable	6 (13.3%)	7 (9.1%)	13 (9.8%)	
Rearrangement				0.083^2^
Single hit	20 (35.7%)	18 (23.4%)	38 (28.6%)	
Double/triple hit	26 (46.4%)	51 (66.2%)	77 (57.9%)	
Missing *BCL2*/*BCL6*	10 (17.9%)	8 (10.4%)	18 (13.5%)	
Days before start treatment				0.317^a^
Median	15.0	19.0	17.0	
IQR	10.8–23.8	11.0–26.0	11.0–26.0	
Range	5.0–84.0	0.0–69.0	0.0–84.0	
Response				0.556^c^
Complete remission	37 (69.8%)	62 (80.5%)	99 (76.2%)	
Partial remission	11 (20.8%)	11 (14.3%)	22 (16.9%)	
Stable disease	1 (1.9%)	1 (1.3%)	2 (1.5%)	
Progressive disease	4 (7.5%)	3 (3.9%)	7 (5.4%)	
(Missing)	3	0	3	
Response (grouped)				0.209^b^
Complete remission	37 (69.8%)	62 (80.5%)	99 (76.2%)	
No complete remission	16 (30.2%)	15 (19.5%)	31 (23.8%)	
(Missing)	3	0	3	

^a^Kruskal–Wallis rank sum test.

^b^Fisher’s Exact Test for Count Data.

^c^Trend test for ordinal variables.

### Overall survival

**Fig. 2:**
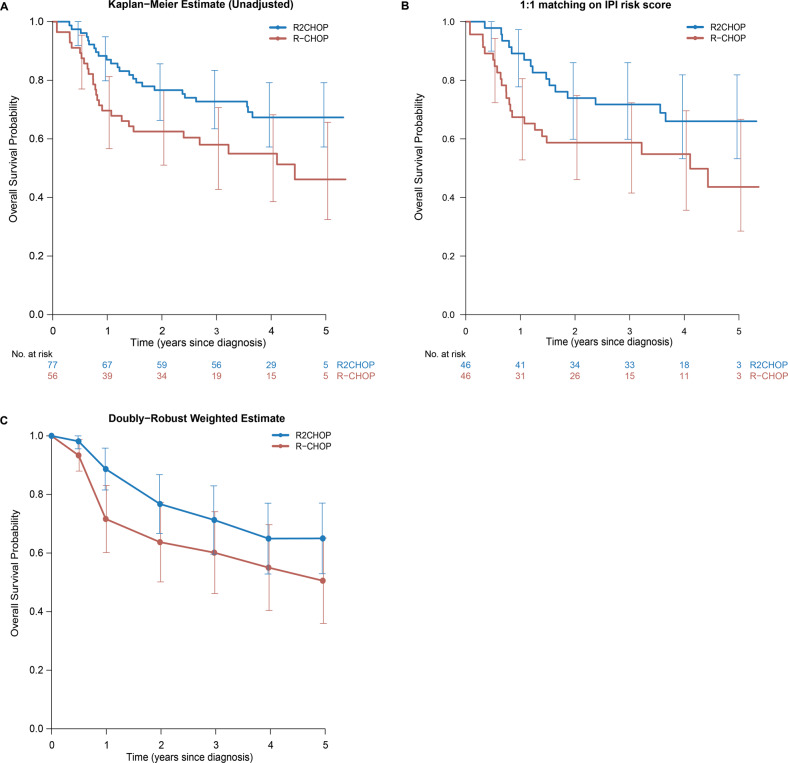
Overall survival analysis in *MYC*-R patients. Overall survival analysis in *MYC*-R patients treated with R2CHOP in blue versus R-CHOP in red in **A** an unadjusted comparison of the overall survival by treatment, **B** comparison of the overall survival in the patients one-to-one matched on IPI risk score, and **C** doubly robust analysis using AIPTW with IPCW estimate of overall survival. Error bars represent 95% confidence interval.

The unadjusted OS of the patients treated with R2CHOP was significantly longer than in the R-CHOP cohort with a hazard ratio (HR) of 0.54 (95% CI 0.31–0.94, *p* = 0.031; Fig. 2A). To reduce bias resulting from baseline imbalances between the cohorts, we applied the three statistical methods described in the methods section.

First, we performed an analysis of the patients who were matched on IPI score. For this analysis, 46 pairs could be analyzed (Table 2) and an identical HR of 0.53 (95% CI 0.28–1.03, *p* = 0.061; Fig. 2B) was found.

**Table 2:** Analysis in patients matched on IPI risk score.

	R-CHOP (*N* = 46)	R2CHOP (*N* = 46)	Total (*N* = 92)	*p*-value
Age at incidence (years)				0.110^a^
Median	70	65	68	
IQR	57–76	58–72	57–75	
Range	29–88	28–82	28–88	
Sex				0.829^b^
Male	28 (60.9%)	30 (65.2%)	58 (63.0%)	
Female	18 (39.1%)	16 (34.8%)	34 (37.0%)	
Ann Arbor stage				0.258^c^
2	9 (19.6%)	10 (21.7%)	19 (20.7%)	
3	11 (23.9%)	5 (10.9%)	16 (17.4%)	
4	26 (56.5%)	31 (67.4%)	57 (62.0%)	
WHO performance score				0.167^c^
0	19 (43.2%)	30 (65.2%)	49 (54.4%)	
1	14 (31.8%)	11 (23.9%)	25 (27.8%)	
2	8 (18.2%)	4 (8.7%)	12 (13.3%)	
3	3 (6.8%)	1 (2.2%)	4 (4.4%)	
(Missing)	2	0	2	
WHO PS (grouped)				0.083^c^
0	19 (43.2%)	30 (65.2%)	49 (54.4%)	
1	14 (31.8%)	11 (23.9%)	25 (27.8%)	
2 or 3	11 (25.0%)	5 (10.9%)	16 (17.8%)	
(Missing)	2	0	2	
LDH				1.000^b^
Within normal range	13 (28.3%)	13 (28.3%)	26 (28.3%)	
Elevated	33 (71.7%)	33 (71.7%)	66 (71.7%)	
Extra-nodal localizations				0.536^c^
None	11 (23.9%)	13 (28.3%)	24 (26.1%)	
1	17 (37.0%)	12 (26.1%)	29 (31.5%)	
2 or more	18 (39.1%)	21 (45.7%)	39 (42.4%)	
IPI risk group				0.836^c^
Low	9 (19.6%)	9 (19.6%)	18 (19.6%)	
Low-intermediate	8 (17.4%)	11 (23.9%)	19 (20.7%)	
High-intermediate	13 (28.3%)	10 (21.7%)	23 (25.0%)	
High	16 (34.8%)	16 (34.8%)	32 (34.8%)	
IPI risk (3 Groups)				1.000^c^
Low	9 (19.6%)	9 (19.6%)	18 (19.6%)	
Intermediate	21 (45.7%)	21 (45.7%)	42 (45.7%)	
High	16 (34.8%)	16 (34.8%)	32 (34.8%)	
Rearrangement				0.113^b^
Single hit	14 (30.4%)	15 (32.6%)	29 (31.5%)	
Double/triple hit	22 (47.8%)	28 (60.9%)	50 (54.3%)	
Missing BCL2/BCL6	10 (21.7%)	3 (6.5%)	13 (14.1%)	
Days before start treatment				0.072^a^
Median	15.5	21.5	19.0	
IQR	12.0–25.2	15.2–27.8	13.0–27.0	
Range	5.0–84.0	2.0–69.0	2.0–84.0	
Response				0.351^c^
Complete remission	29 (67.4%)	38 (82.6%)	67 (75.3%)	
Partial remission	10 (23.3%)	6 (13.0%)	16 (18.0%)	
Stable disease	1 (2.3%)	0 (0.0%)	1 (1.1%)	
Progressive disease	3 (7.0%)	2 (4.3%)	5 (5.6%)	
(Missing)	3	0	3	

^a^Kruskal–Wallis rank sum test.

^b^Fisher’s Exact Test for Count Data.

^c^Trend test for ordinal variables.

Second, in multivariable analysis, adjusting for the variables sex, age at diagnosis, Ann Arbor stage, number of extra-nodal localizations, LDH, WHO performance status, and rearrangement status (Table 3), yielded a comparable HR of 0.51 (95% CI 0.26–1.00, *p* = 0.049).

**Table 3:** Multivariable Cox proportional-hazards regression for overall survival.

Variable		HR	95%CI	*p*-value
Treatment	R-CHOP	1		
	R2CHOP	0.51	(0.26–1.00)	0.049
Sex	Male	1		
	Female	0.55	(0.29–1.06)	0.074
Age at incidence (years)		1.03	(1.00–1.06)	0.025
Ann Arbor stage	2	1		
	3	0.50	(0.15–1.61)	0.24
	4	0.88	(0.29–2.66)	0.82
Extra-nodal localizations	None	1		
	1	0.55	(0.23–1.32)	0.18
	2 or more	0.63	(0.27–1.47)	0.28
LDH	Within normal range	1		
	Elevated	3.96	(1.42–11.06)	0.009
WHO PS (grouped)	0	1		
	1	1.41	(0.68–2.93)	0.36
	2 or 3	2.17	(0.98–4.79)	0.056
Rearrangement	Single hit	1		
	Double/triple hit	0.98	(0.49–1.94)	0.95
	Missing BCL2/BCL6	0.49	(0.16–1.53)	0.22

Third, estimation of the treatment effect of R2CHOP over R-CHOP on the total cohort by means of IPTW resulted in a HR of 0.59 (95% CI of 0.32–1.10, *p* = 0.10), and the absolute estimates using the doubly robust method are shown in Fig. 2C. Assessments of the common support assumption and the reduction of imbalance are presented in the supplementary data (Table S2 and Fig. S1).

### Progression-free survival

**Fig. 3:**
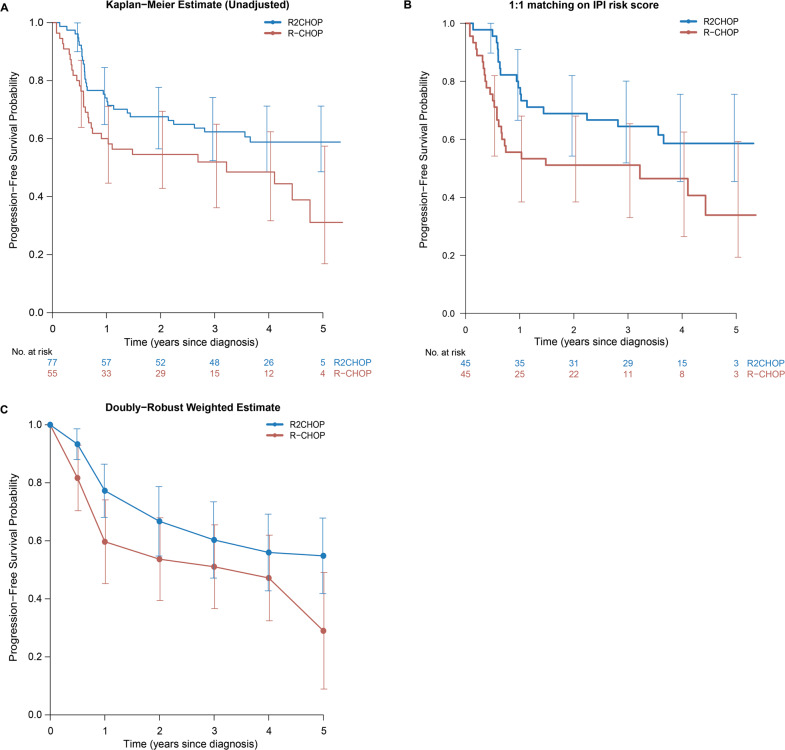
Progression-free survival analysis in *MYC*-R patients. Progression-free survival analysis in *MYC*-R patients treated with R2CHOP in blue versus R-CHOP in red in **A** an unadjusted comparison of the overall survival by treatment, **B** comparison of the progression-free survival in the patients one-to-one matched on IPI risk score and **C** doubly robust analysis using AIPTW with IPCW estimate of progression-free survival. Error bars represent 95% confidence interval.

The unadjusted HR of PFS was 0.60 (95% CI 0.36–0.99, *p* = 0.045) in favor of R2CHOP (Fig. 3A).

We analyzed the PFS using the same methods as for OS. In the set matched on IPI score a HR of 0.53 (95% CI 0.29–0.97, *p* = 0.039; Fig. 3B) was found. Multivariable analysis resulted in a comparable HR of 0.59 (95% CI 0.32–1.06, *p* = 0.075, Table S3). In the weighted analysis (ITPW), the HR was 0.60 (95% CI 0.32–1.12, *p* = 0.11) (Fig. 3C).

### Overall survival and progression-free survival by rearrangement status

**Fig. 4:**
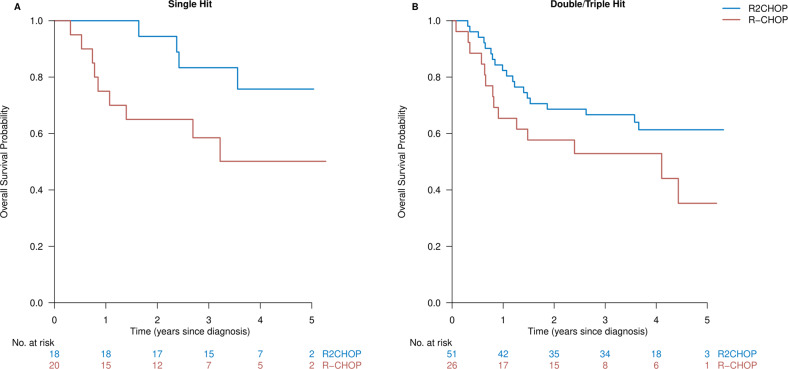
Subgroup analysis of overall survival per rearrangement status. *MYC*-R patients treated with R2CHOP in blue versus R-CHOP in red in an unadjusted comparison depicted for **A** single-hit patients and **B** double/triple-hit patients.

**Fig. 5:**
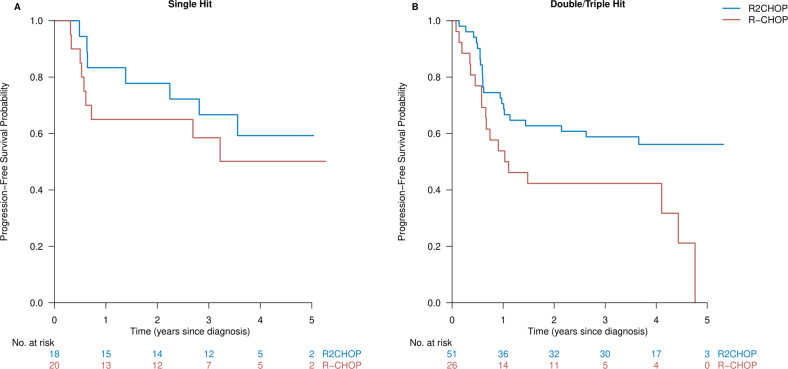
Subgroup analysis of progression-free survival per rearrangement status. *MYC*-R patients treated with R2CHOP in blue versus R-CHOP in red in an unadjusted comparison depicted for **A** single-hit patients and **B** double/triple-hit patients.

As rearrangement status (SH or DH/TH) is known to be of prognostic importance for survival (although it was not statistically significant in our dataset, Table 1), we did a subgroup analysis. Without any covariate adjustment, both SH and DH/TH patients tended to have a longer OS when treated with R2CHOP than when treated with R-CHOP with a HR of 0.34 (95% CI of 0.10–1.10, *p* = 0.072) (Fig. 4A) and a HR of 0.57 (95% CI of 0.28–1.13, *p* = 0.11) (Fig. 4B), respectively. For PFS, the HRs were 0.66 in the SH subgroup (95% CI of 0.25–1.79, *p* = 0.42) (Fig. 5A) and 0.48 in the DH/TH subgroup (95% CI of 0.26–0.90, *p* = 0.022) (Fig. 5B). There were baseline imbalances within the subgroups (Table S4), but we were unable to adjust properly due to low patient numbers.

## Discussion

To date, no published randomized trials were able to demonstrate improvements in overall survival over induction treatment with R-CHOP for patients with *MYC*-R DLBCL. Here, we present a comparison of addition of lenalidomide to R-CHOP (R2CHOP) versus R-CHOP as first-line treatment for newly diagnosed *MYC*-R DLBCL. We used long-term follow-up data of patients treated with R2CHOP in the single-arm phase-II HOVON-130 trial [18]. The analysis was extended by adding a cohort of patients who were treated with R-CHOP and met the inclusion criteria of the study, but were either not invited for logistic reasons, or who declined to participate in the trial. As the two treatment regimens in this analysis were not randomized, any direct comparison is subject to treatment-selection bias due to systematic differences between the characteristics of the patients in the two groups. Therefore, we used three statistical methods (1:1 matching, multivariable analysis and weighting using the propensity score) to reduce treatment-selection bias. Our three methods consistently showed improved survival after R2CHOP with HRs of approximately 0.59 for OS and 0.60 for PFS.

Using a propensity score model is an upcoming method in clinical cancer research and has been applied to other lymphoma trials [20–22]. A major strength of this method is the possibility to adjust for large numbers of variables and obtain a similar distribution of baseline variables among two treatment groups. The most important limitation of weighting using the propensity score is that the propensity score has to be estimated using a statistical model, and it is impossible to verify whether this model was correctly specified [23]. To circumvent this limitation, we used a doubly robust method. However, only a large randomized controlled trial (RCT) can balance observed as well as unobserved characteristics. Furthermore, the treatment-effect estimate from this method was not statistically significant at the commonly accepted significance level of 5%. Therefore, the results presented here do not provide the same level of evidence that addition of lenalidomide is superior to R-CHOP alone as a confirmatory phase 3 study.

Of the three statistical methods that we used, in theory weighting using the propensity score most closely resembles the result of an RCT and has the advantage that it estimates the average treatment effect on the entire sample. Hence, the current propensity comparison of R2CHOP versus R-CHOP is a valuable alternative for a RCT. The effect on overall survival found here, with a HR of 0.59 as the most conservative estimate from the three methods used, and despite the relatively small sample size, the stability of the HRs for OS and PFS across the three methods lends credibility to the conclusion of a survival benefit for *MYC*-R patients treated with R2CHOP.

In the subgroups determined by rearrangement status we performed unadjusted OS and PFS analysis, because we were not able to adjust for baseline imbalances because of the low number of events within the subgroups. These analyses can therefore not be interpreted as estimations of the effect of lenalidomide. However, combined with the results of the multivariable analysis of the total cohort, they can be interpreted as an indication that the treatment effect is consistent across the rearrangement subgroups. Notably, CNS localization and with HIV positivity were exclusion criteria in the HOVON-130 trial. Consequently, whether patients in one subgroup benefit more from R2CHOP than another, as well as the effect of R2CHOP on patients with CNS lymphoma or HIV positivity, needs to be investigated in future, larger studies.

The use of a nationwide observational cohort as control arm for the current enables us to compare of R2CHOP versus R-CHOP as first-line treatment for *MYC*-R DLBCL patients. Nonetheless, baseline differences have to be critically addressed. In our cohort, the WHO performance status reflects that patients in the R-CHOP group are clinically less fit than patients in the R2CHOP group. This could be due to the fact that patients from the HOVON-900 cohort that were treated with other treatment regimens than R-CHOP (i.e., no R-CHOP, more intensive regimens or less intensive regimens) were excluded from this analysis, suggesting that there might have been some selection bias in treating less clinically fit patients with R-CHOP.

The recent discovery that a shorter diagnosis to treatment interval (DTI) associates with an inferior outcome in DLBCL [24], is also applicable on our cohorts (15 days before start treatment in the R-CHOP group and 19 days in the R2CHOP group). However, the time difference of four days was relatively small. The relative delay in DTI in the R2CHOP group is most likely due to study-related work-up.

Two previous phase II studies showed that addition of lenalidomide to R-CHOP is effective in newly diagnosed DLBCL patients [25, 26]. Both studies report that R2CHOP was particular effective in patients with the non-GCB (or ABC) subtype. However, the larger phase III ROBUST trial, in which only newly diagnosed ABC type DLBCL patients were included and randomized for R2CHOP or R-CHOP, did not meet the primary end point of superior PFS for R2CHOP treated patients [27].

Although the non-GCB subtype is generally associated with inferior survival outcomes, heterogeneity between the COO subtypes remain and recent studies provided additional insights in the complex genomic landscape of DLBCL [28–30]. Besides, the afore mentioned studies did not distinguish for *MYC* rearrangement status. A *MYC* translocation is most common in the GCB subtype. In line with this, the GCB subtype is overrepresented in our cohorts. We hypothesize that R2CHOP in our cohort of *MYC*-R patients with mainly a GCB subtype is effective largely due to the MYC-downregulating effects of lenalidomide. We do not exclude the possibility that lenalidomide has different mechanism of action in the ABC subtype or other molecular and genomic DLBCL subtypes.

We previously showed in the primary end-point analysis of the HOVON-130 study that the addition of lenalidomide to R-CHOP is well-tolerated and has limited and manageable adverse effects. There were no treatment-related deaths, and fewer grade 3 infections than expected for the more intensive immunochemotherapy regimen DA-EPOCH-R [18]. R2CHOP can be fully given on an outpatient basis, which is a major advantage to patients’ well-being, does not need a central venous catheter and requires less hospital admission days and is associated with fewer (serious) adverse effects. Besides, the sharp drop in the price of lenalidomide in the EU since March 2022 after the patent expiration on lenalidomide makes R2CHOP treatment likely to be cost-effective.

For current clinical practice R2CHOP is incorporated as an alternative option for DA-EPOCH-R in the Dutch guideline for patients with *MYC*-R DLBCL. Future studies may explore whether adding next-generation cereblon modulating antigens to R-CHOP can further increase the effects we observed here.

## Supplementary information

R2CHOP vs R-CHOP in MYC-R DLBCL - supplements

## Data Availability

The data that support the findings of this study are available from HOVON and the Netherlands Cancer Registry (NCR) but restrictions apply to the availability of these data, which were used under license for the current study, and so are not publicly available. Data are however available from the authors upon reasonable request and with permission of HOVON and the Netherlands Cancer Registry (NCR).
