# Supplementary material for: A non-randomized risk-adjusted comparison of lenalidomide + R-CHOP versus R-CHOP for MYC-rearranged DLBCL patients
Source: Blood Cancer J. 2023 May 22;13(1):85. doi: 10.1038/s41408-023-00854-2 (PMC10203347; doi:10.1038/s41408-023-00854-2)
Supplement: Supplementary file 1 — R2CHOP vs R-CHOP in MYC-R DLBCL - supplements [file 41408_2023_854_MOESM1_ESM.docx]

**A propensity score-adjusted comparison of lenalidomide + R-CHOP versus R-CHOP for *MYC*-rearranged DLBCL patients**

**Supplementary material**

[Supplementary Methods 1](#_Toc125716902)

[Proportional hazards regression 1](#_Toc125716903)

[Matching 2](#_Toc125716904)

[Inverse probability of treatment weighting (IPTW) 2](#_Toc125716905)

[Missing values 3](#_Toc125716906)

[MYC-R patients from HOVON-900 not meeting eligibility criteria 4](#_Toc125716907)

[Table S1 MYC-R patients of HOVON-900 not meeting eligibility criteria 4](#_Toc125716908)

[Propensity Score Diagnostics 5](#_Toc125716909)

[Table S2. Baseline Imbalances Before and After Weighting 5](#_Toc125716910)

[Progression-Free Survival 6](#_Toc125716911)

[Table S3. Multivariable Cox proportional-hazards regression 6](#_Toc125716912)

[Baseline Characteristics by Rearrangement Subgroup 7](#_Toc125716913)

[Table S4. Baseline characteristics by rearrangement subgroup 7](#_Toc125716914)

[Histograms of the Propensity Scores 9](#_Toc125716915)

[Figure S1. Histograms of the distribution of the propensity scores per treatment group. 9](#_Toc125716916)

[Regulatory Information 10](#_Toc125716917)

[References of Supplementary Methods 10](#_Toc125716918)

# Supplementary Methods

## Proportional hazards regression

The proportional hazards assumption was checked using a score test, which was valid for all variables, except possibly for the parameter that belonged to the patients with missing *BCL2*/*BCL6* status (p=0.02 in multivariable analysis of overall survival). For this category the relative hazard (compared with single-hit patients) decreased over time. However, this category is not of interest in itself, but was created so that patients with missing rearrangement status could be kept in the model. Therefore, it was assumed that the non-proportionality of this variable is of little consequence for the hazard ratio (HR) of the treatment effect^1^. The HR from the multivariable regression is an adjusted HR and can be interpreted as the ratio of the hazards for two hypothetical patients whose only difference is that one was treated with R2CHOP, and the other with R-CHOP. The hazard ratio is non-collapsible, which means that the adjusted HR is not the same as the unadjusted HR, even if the treatment assignment (R2CHOP versus R-CHOP) would have been completely random. A consequence of this non-collapsibility property of the HR, is that the HR estimated by the multivariable regression here is not the same as the HR that would have been found in an unadjusted analysis of a randomized trial. The HR of the multivariable regression is called the “conditional treatment effect”, while the HR of a randomized trial is the “average treatment effect” (ATE).

## Matching

Matching was performed without replacement^2^, meaning that any patient treated with R-CHOP was only used once as a match for a patient treated with R2CHOP. Patients (from either group) were discarded if they had no available matches with the same IPI score (grouped as low, intermediate, or high), or if the set of their potential matches was depleted. By doing so, the IPI-risk distribution in the resulting matched dataset was artificially constructed to be equal to its distribution in the R2CHOP group. A limitation hereof is that the HR from this analysis estimates the treatment effect on patients treated with R2CHOP in this particular sample. This is called the “average treatment effect on the treated” (ATT). The ATT does not adequately estimate the ATE in the target population, which consists of all patients (i.e. both groups combined) in the present analysis.

## Inverse probability of treatment weighting (IPTW)

Inverse probability of treatment weighting (IPTW) was applied using a propensity score. This is a method to estimate the ATE in the target population. These HRs are, therefore, most likely to reflect what would have been observed in an unadjusted randomized comparison, in contrast to multivariable analysis and matching. An advantage of IPTW over matching methods, is that IPTW uses the entire sample and does not need to remove patients for whom no match can be found.

We calculated a propensity score for being included in the HOVON-130 trial which was estimated from a logistic model based on the separate components of the IPI risk score, i.e., age, Ann Arbor stage, WHO performance score, number of extra-nodal localizations, serum LDH level. We additionally included sex and rearrangement status (single hit versus double/triple hit) in the model because these are known prognostic factors for overall survival. Patients with missing values were removed, except patients with unknown *BCL2*/*BCL6* rearrangement status; these were considered as a separate category. The propensity score was used for ITPW, where the IPTW weights were truncated at the 5-th and 95-th percentiles of their distribution. Standardized differences of baseline variables were calculated before and after weighting to assess the reduction of imbalances. The common support assumption was verified using the distribution of the propensity score across the treatment groups (Supplementary Figure S1). To allow for some degree of misspecification of the model for the propensity score, we did a separate analysis using the augmented IPTW (AIPTW) estimator, which is a doubly-robust augmented IPTW estimator that combines inverse probability-of-censoring weighting (IPCW) and the g-formula^3^. It is a doubly-robust estimator, which means that it is consistent if either the outcome model (for OS or for PFS), or the propensity model is correctly specified (but not if both are incorrect). Models for the treatment propensity, censoring, and outcome were specified using the same variables as used to estimate the propensity score (with actual treatment received also as variable in the latter two models). Thereby, we obtained absolute estimates of the ATE at 6 months and years 1 up to 5 since diagnosis. However, this method is not suited to estimate hazard ratios.

Analyses were performed in R version 4.1.2 with package riskRegression version 2021.10.10.

## Missing values

The IPI score was calculated using the prognostic factors that it consists of (Age, Ann Arbor stage, WHO performance status, serum LDH level, and number of extra-nodal sites). If a missing value in these prognostic factors meant that the IPI score could not be calculated, it was set to missing. However, if for particular patients the presence of the risk factor would not change the score, the score was not considered missing. For example, if the performance status was missing but the patient had none of the other risk factors, the IPI risk group was set to low.

Patients with missing IPI risk category were removed from the IPI-matched analysis.

The same principle was used for the categorization of the rearrangement status. This variable was constructed using the BCL2/BCL6 information. Rearrangement status was considered missing if BCL2 or BCL6 measurement on FISH was missing, except if it could be determined for certain despite the missing values. For example, a *MYC*-R patient with a BCL2 rearrangement a but BCL6 missing, was counted in the double/triple hit category.

Missing rearrangement status was used as a separate category in the propensity score model and multivariable proportional hazards regression. In the subgroup analysis of rearrangement status however, patients whose category (single hit, double/triple hit) could not unambiguously be determined were removed from the analysis.

Patients with missing values in other variables that were needed in the multivariable model or to calculate the propensity score, were removed from these particular analyses.

# MYC-R patients from HOVON-900 not meeting eligibility criteria

## Table S1 MYC-R patients of HOVON-900 not meeting eligibility criteria

|  | Number of patients (%) |
| --- | --- |
| **MYC-R patients from HOVON-900 not meeting eligibility criteria** | **48** |
| Reason ineligible |  |
| No treatment | 17 (35.4%) |
| No R-CHOP  DA-EPOCH-R  R-miniCHOP  R-CEOP  Inclusion in other trial (HOVON-151)  MISSING | 16 (33.3%)  8 (50% from 16)  4 (25% from 16)  1 (6,25% from 16)  2 (12,5% from 16)  1 (6,25% from 16) |
| Ann Arbor stage I | 15 (31.1%) |

# Propensity Score Diagnostics

## Table S2. Baseline Imbalances Before and After Weighting

| Variable or category | Standardized difference | Standardized difference  after weighting |
| --- | --- | --- |
| Age | 0.3711 | 0.0617 |
| Sex Female | 0.1578 | 0.0034 |
| Ann Arbor stage 3 | 0.2521 | 0.1017 |
| Ann Arbor stage 4 | -0.4049 | -0.1408 |
| WHO PS 1 | -0.0298 | 0.0059 |
| WHO PS 2 or 3 | 0.57 | 0.1047 |
| LDH Elevated | -0.116 | -0.0322 |
| 1 Extranodal localization | 0.2864 | 0.0756 |
| 2 Or more extranodal localizations | -0.1148 | -0.0802 |
| Double/triple hit | -0.4497 | -0.0645 |
| Missing BCL2/BCL6 status | 0.2406 | 0.0584 |

# Progression-Free Survival

## Table S3. Multivariable Cox proportional-hazards regression

| **Variable** |  | **HR** | **95%CI** | **p-value** |
| --- | --- | --- | --- | --- |
| Treatment | R-CHOP | 1 |  |  |
|  | R2CHOP | 0.59 | (0.32–1.06) | 0.075 |
| Sex | Male | 1 |  |  |
|  | Female | 0.52 | (0.29–0.94) | 0.03 |
| Age at incidence (years) |  | 1.01 | (0.99–1.04) | 0.31 |
| Ann Arbor stage | 2 | 1 |  |  |
|  | 3 | 0.65 | (0.24–1.77) | 0.40 |
|  | 4 | 0.95 | (0.36–2.49) | 0.91 |
| Extranodal localizations | None | 1 |  |  |
|  | 1 | 0.72 | (0.33–1.57) | 0.40 |
|  | 2 or more | 0.59 | (0.27–1.30) | 0.19 |
| LDH | Within normal range | 1 |  |  |
|  | Elevated | 3.21 | (1.40–7.38) | 0.006 |
| WHO PS (grouped) | 0 | 1 |  |  |
|  | 1 | 1.63 | (0.84–3.15) | 0.15 |
|  | 2 or 3 | 2.00 | (0.95–4.18) | 0.066 |
| Rearrangement | Single hit | 1 |  |  |
|  | Double/triple hit | 1.00 | (0.54–1.85) | 0.99 |
|  | Missing BCL2/BCL6 | 0.49 | (0.17–1.38) | 0.18 |

# Baseline Characteristics by Rearrangement Subgroup

## Table S4. Baseline characteristics by rearrangement subgroup

|  | **Single Hit** | | **Double/Triple Hit** | |
| --- | --- | --- | --- | --- |
|  | R-CHOP (N=20) | R2CHOP  (N=18) | R-CHOP (N=26) | R2CHOP  (N=51) |
| **Age** |  |  |  |  |
| Median | 67 | 62 | 72 | 65 |
| IQR | 58–72 | 57–70 | 59–75 | 54–72 |
| Range | 29 - 88 | 30 - 77 | 38 - 84 | 28 - 82 |
| **Sex** |  |  |  |  |
| Male | 14 (70.0%) | 14 (77.8%) | 16 (61.5%) | 34 (66.7%) |
| Female | 6 (30.0%) | 4 (22.2%) | 10 (38.5%) | 17 (33.3%) |
| **Ann Arbor stage** |  |  |  |  |
| 2 | 3 (15.0%) | 4 (22.2%) | 7 (26.9%) | 6 (11.8%) |
| 3 | 5 (25.0%) | 1 (5.6%) | 7 (26.9%) | 8 (15.7%) |
| 4 | 12 (60.0%) | 13 (72.2%) | 12 (46.2%) | 37 (72.5%) |
| **WHPO PS** |  |  |  |  |
| 0 | 8 (42.1%) | 13 (72.2%) | 11 (45.8%) | 30 (58.8%) |
| 1 | 7 (36.8%) | 3 (16.7%) | 5 (20.8%) | 17 (33.3%) |
| 2 | 3 (15.8%) | 2 (11.1%) | 4 (16.7%) | 3 (5.9%) |
| 3 | 1 (5.3%) | 0 (0.0%) | 4 (16.7%) | 1 (2.0%) |
| (Missing) | 1 | 0 | 2 | 0 |
| **WHO PS (grouped)** |  |  |  |  |
| 0 | 8 (42.1%) | 13 (72.2%) | 11 (45.8%) | 30 (58.8%) |
| 1 | 7 (36.8%) | 3 (16.7%) | 5 (20.8%) | 17 (33.3%) |
| 2 or 3 | 4 (21.1%) | 2 (11.1%) | 8 (33.3%) | 4 (7.8%) |
| (Missing) | 1 | 0 | 2 | 0 |
| **LDH** |  |  |  |  |
| Within normal range | 8 (40.0%) | 7 (41.2%) | 6 (23.1%) | 10 (19.6%) |
| Elevated | 12 (60.0%) | 10 (58.8%) | 20 (76.9%) | 41 (80.4%) |
| (Missing) | 0 | 1 | 0 | 0 |
| **Extranodal** |  |  |  |  |
| None | 4 (20.0%) | 3 (16.7%) | 7 (26.9%) | 17 (33.3%) |
| 1 | 10 (50.0%) | 9 (50.0%) | 11 (42.3%) | 10 (19.6%) |
| 2 or more | 6 (30.0%) | 6 (33.3%) | 8 (30.8%) | 24 (47.1%) |
| **IPI Risk Group** |  |  |  |  |
| Low | 5 (26.3%) | 5 (29.4%) | 6 (23.1%) | 4 (7.8%) |
| Low-intermediate | 4 (21.1%) | 4 (23.5%) | 3 (11.5%) | 15 (29.4%) |
| High-intermediate | 4 (21.1%) | 4 (23.5%) | 6 (23.1%) | 20 (39.2%) |
| High | 6 (31.6%) | 4 (23.5%) | 11 (42.3%) | 12 (23.5%) |
| (Missing) | 1 | 1 | 0 | 0 |
| **IPI Risk (3 Groups)** |  |  |  |  |
| Low | 5 (26.3%) | 5 (27.8%) | 6 (23.1%) | 4 (7.8%) |
| Intermediate | 8 (42.1%) | 9 (50.0%) | 9 (34.6%) | 35 (68.6%) |
| High | 6 (31.6%) | 4 (22.2%) | 11 (42.3%) | 12 (23.5%) |
| (Missing) | 1 | 0 | 0 | 0 |
| **Response** |  |  |  |  |
| Complete remission | 13 (68.4%) | 16 (88.9%) | 17 (68.0%) | 40 (78.4%) |
| Partial remission | 4 (21.1%) | 1 (5.6%) | 5 (20.0%) | 8 (15.7%) |
| Stable disease | 1 (5.3%) | 0 (0.0%) | 0 (0.0%) | 1 (2.0%) |
| Progressive diease | 1 (5.3%) | 1 (5.6%) | 3 (12.0%) | 2 (3.9%) |
| (Missing) | 1 | 0 | 1 | 0 |

# Histograms of the Propensity Scores

## Figure S1. Histograms of the distribution of the propensity scores per treatment group.


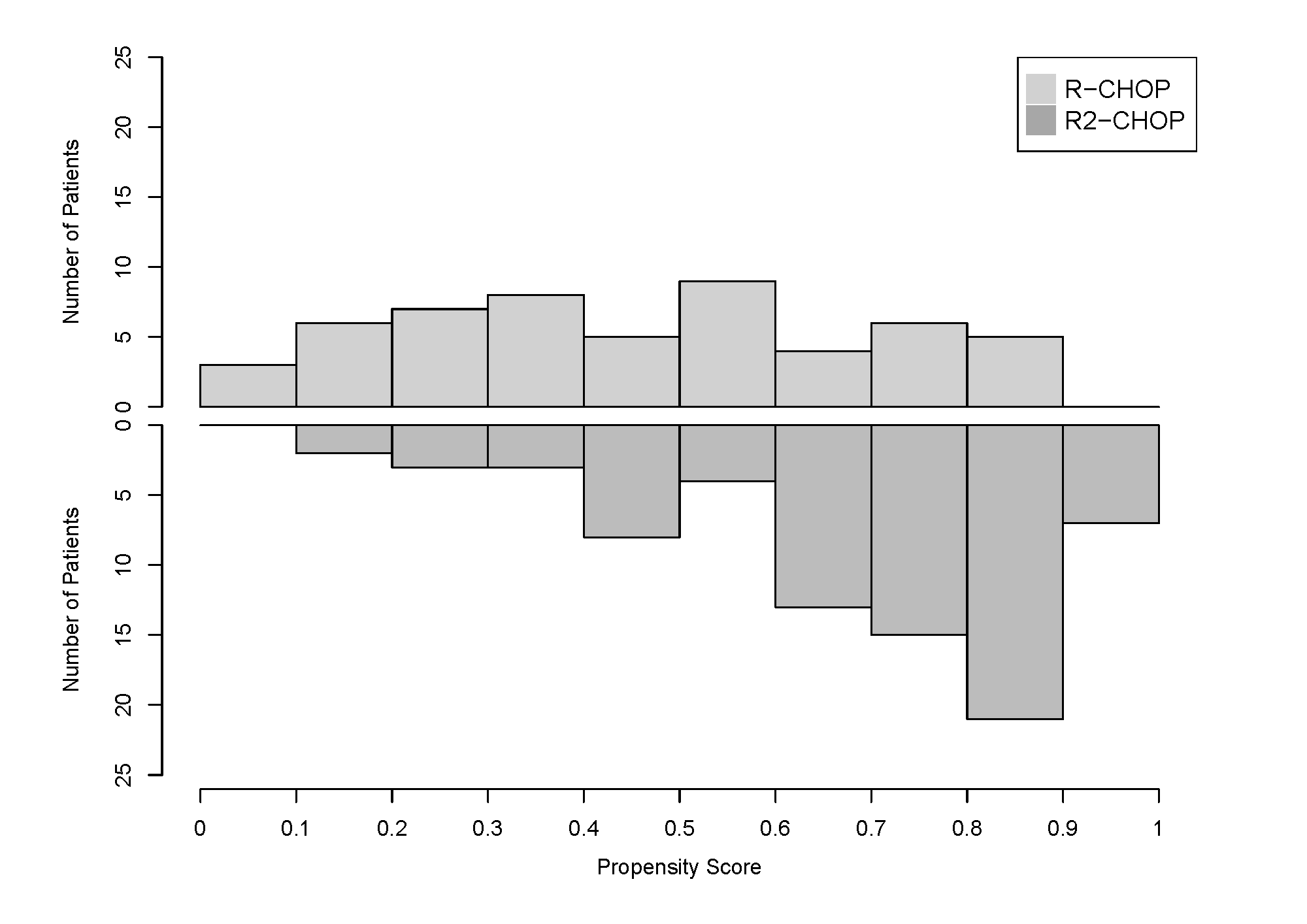


# Regulatory Information

The HOVON-130 trial (Eudra-CT 2014-002654-39) and the HOVON-900 cohort were approved by the medical ethics committee (METC) of the Amsterdam UMC (METC VUMC 2015.082). Patients gave written informed consent to participate. The NCR Privacy Review Board additionally approved the use of de-identified data for this study.

# References of Supplementary Methods

1. Schemper M. Cox Analysis of Survival Data with Non-Proportional Hazard Functions. *Journal of the Royal Statistical Society Series D (The Statistician)*. 1992;41(4):455-465.

2. Austin PC. An Introduction to Propensity Score Methods for Reducing the Effects of Confounding in Observational Studies. *Multivariate Behav Res*. May 2011;46(3):399-424. doi:10.1080/00273171.2011.568786

3. Ozenne BMH, Scheike TH, Staerk L, Gerds TA. On the estimation of average treatment effects with right-censored time to event outcome and competing risks. *Biom J*. May 2020;62(3):751-763. doi:10.1002/bimj.201800298
